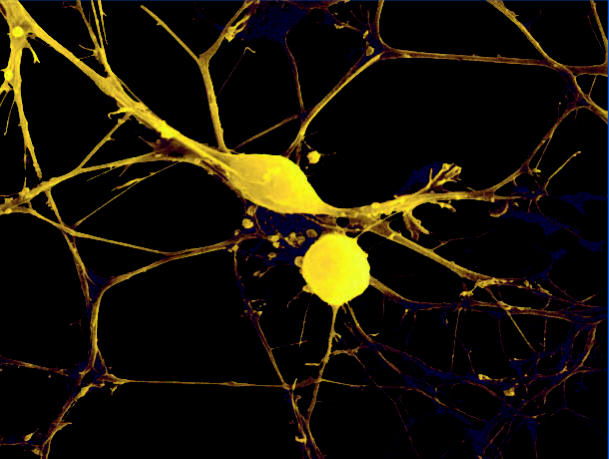# Headliners: Neurodegenerative Disease: Inflammatory Enzyme Modulates Motor Neuron Damage in Amyotrophic Lateral Sclerosis

**Published:** 2006-12

**Authors:** Jerry Phelps

Wu DC, Ré DB, Nagai M, Ischiropoulos H, Przedborski S. 2006. The inflammatory NADPH oxidase enzyme modulates motor neuron degeneration in amyotrophic lateral sclerosis mice. Proc Natl Acad Sci U S A 103:12132–12137.

Approximately 30,000 patients in the United States currently have amyotrophic lateral sclerosis (ALS), also known as Lou Gehrig’s disease. ALS is a progressive neuromuscular disease that weakens and eventually destroys motor neurons that connect the brain with the skeletal muscles. NIEHS grantee Serge Przedborski of Columbia University has pioneered the investigation of the molecular mechanisms leading to the death of neurons that occurs in ALS and Parkinson disease. Now Przedborski and colleagues provide new insights into the role of the enzyme NADPH oxidase in the death of motor neurons as a result of ALS.

ALS, the most common adult-onset paralytic disease, is most commonly diagnosed in middle age, and affects men more often than women. Patients gradually lose the ability to speak, swallow, and move voluntarily. Sensory function and intellectual ability are unaffected, and death usually results from loss of respiratory function. The disease affects all racial, socioeconomic, and ethnic groups, and the life expectancy of ALS patients is usually three to five years after diagnosis.

In this study, the investigators observed spinal cord tissue using a mouse histochemistry model, and created a timeline chronicling observed motor abnormality behavior in transgenic SOD1^G93A^ mice. They also conducted histological evaluations of postmortem human tissue using a control group and an ALS group.

The researchers discovered that the NADPH oxidase enzyme, an important component in the generation of destructive reactive oxygen species during inflammation, is active in the spinal cords of ALS patients and also in the mouse model of the disease. When they inactivated the enzyme in the mice, they found that neurodegeneration was significantly delayed, and the mice lived longer. Additional studies also showed that NADPH oxidase–derived oxidative products also damaged proteins including insulin-like growth factor 1 receptors located on motor neurons.

Insulin-like growth factor 1 has been demonstrated to have therapeutic potential in ALS patients. The authors conclude that these results suggest that coadministration of an antiinflammatory agent with the protein may improve its efficacy in ALS patients.

## Figures and Tables

**Figure:**